# Lipid kinases PIP5K7 and PIP5K9 are required for polyamine‐triggered K^+^ efflux in Arabidopsis roots

**DOI:** 10.1111/tpj.14932

**Published:** 2020-08-19

**Authors:** Xavier Zarza, Ringo Van Wijk, Lana Shabala, Anna Hunkeler, Matthew Lefebvre, Antia Rodriguez‐Villalón, Sergey Shabala, Antonio F. Tiburcio, Ingo Heilmann, Teun Munnik

**Affiliations:** ^1^ Research Cluster Green Life Sciences Section Plant Cell Biology Swammerdam Institute for Life Sciences University of Amsterdam PO Box 94215 Amsterdam 1090 GE The Netherlands; ^2^ Tasmanian Institute of Agriculture University of Tasmania Hobart Australia; ^3^ Department of Biology Institute of Agricultural Science Swiss Federal Institute of Technology in Zurich Zurich Switzerland; ^4^ International Research Centre for Environmental Membrane Biology Foshan University Foshan China; ^5^ Dept. of Natural Products, Plant Biology and Soil Science University of Barcelona Barcelona Spain; ^6^ Dept of Cellular Biochemistry Institute of Biochemistry and Biotechnology Martin Luther University Halle‐Wittenberg Halle (Saale) Germany

**Keywords:** Arabidopsis, phosphoinositide signalling, phosphatidylinositol 4,5‐bisphosphate (PIP2), phosphatidylinositol 4‐phosphate 5‐kinase (PIP5K), phosphatidic acid (PA), phospholipids, polyamines, K^+^ flux

## Abstract

Polyamines, such as putrescine, spermidine and spermine (Spm), are low‐molecular‐weight polycationic molecules present in all living organisms. Despite their implication in plant cellular processes, little is known about their molecular mode of action. Here, we demonstrate that polyamines trigger a rapid increase in the regulatory membrane lipid phosphatidylinositol 4,5‐bisphosphate (PIP_2_), and that this increase is required for polyamine effects on K^+^ efflux in Arabidopsis roots. Using *in vivo* ^32^P_i_‐labelling of Arabidopsis seedlings, low physiological (μm) concentrations of Spm were found to promote a rapid PIP_2_ increase in roots that was time‐ and dose‐dependent. Confocal imaging of a genetically encoded PIP_2_ biosensor revealed that this increase was triggered at the plasma membrane. Differential ^32^P_i_‐labelling suggested that the increase in PIP_2_ was generated through activation of phosphatidylinositol 4‐phosphate 5‐kinase (PIP5K) activity rather than inhibition of a phospholipase C or PIP_2_ 5‐phosphatase activity. Systematic analysis of transfer DNA insertion mutants identified *PIP5K7* and *PIP5K9* as the main candidates involved in the Spm‐induced PIP_2_ response. Using non‐invasive microelectrode ion flux estimation, we discovered that the Spm‐triggered K^+^ efflux response was strongly reduced in *pip5k7 pip5k9* seedlings. Together, our results provide biochemical and genetic evidence for a physiological role of PIP_2_ in polyamine‐mediated signalling controlling K^+^ flux in plants.

## Significance Statement

Polyamines, such as putrescine, spermidine and spermine, are low‐molecular‐weight polycations present in all living organisms. Despite their involvement in various plant cellular processes, little is known about their molecular mode of action. Here, we demonstrate that polyamines trigger a rapid increase in the signalling lipid PIP_2_ at the plasma membrane of Arabidopsis roots by activating two lipid kinases, PIP5K7 and PIP5K9, and that this lipid response is required for the K^+^ efflux that is triggered downstream.

## INTRODUCTION

Development and adaptation of plants to a changing environment involve numerous cellular signalling pathways. So far, the interactions between signalling pathways and their integration are not well understood. The links between biochemical cascades and their hierarchical interplay are an important and current field of research. Our study focuses on the role of phosphatidylinositol 4,5‐bisphosphate (PIP_2_), an important lipid signalling molecule in plants (Munnik and Nielsen, 2011; Heilmann, 2016b; Noack and Jaillais, 2017; Gerth *et al*., 2017a; Colin and Jaillais, 2020). This minor lipid is formed by phosphorylation of phosphatidylinositol 4‐phosphate (PIP), catalysed by the enzyme PIP 5‐kinase (PIP5K). Vascular plants typically contain very low levels of PIP_2_ per gram fresh weight, being 30‐ to 100‐fold lower than in animal cells (Munnik *et al*., 1998a; Meijer and Munnik, 2003). In plants, PIP_2_ can be detected by metabolic *in vivo* labelling using radioactive phosphate (e.g., ^32^P_i_) (Munnik and Zarza, 2013). Alternatively, genetically encoded fluorescent biosensors are available to visualise PIP_2_ in living cells (van Leeuwen *et al*., 2007; Simon *et al*., 2014). In response to salt or heat stress, a clear plasma membrane localisation of PIP_2_ biosensors was previously observed, which coincided with the formation of ^32^P‐PIP_2_ in a time‐ and dose‐dependent fashion (van Leeuwen *et al*., 2007; Simon *et al*., 2014).

The physiological function of PIP_2_ in plants is evident from the phenotypes of Arabidopsis mutants with genetic lesions in *PIP5K* genes. The Arabidopsis genome encodes a relatively large *PIP5K* gene family of 11 members, which can be categorised into two subfamilies (*PIP5K1‐11;* Mueller‐Roeber and Pical, 2002). Reversed genetics revealed that individual PIP5Ks exhibit specific and distinct roles in plant signalling and development. Some PIP5K isoenzymes are expressed in pollen tubes (PIP5K4–6, 10 and 11) (Sousa *et al*., 2008; Ischebeck *et al*., 2008; Zhao *et al*., 2010; Ischebeck *et al*., 2011) or root hairs (PIP5K3 and 4) (Stenzel *et al*., 2008; Kusano *et al*., 2008a; Wada *et al*., 2015) and are involved in polar tip growth. The ubiquitously expressed PIP5K1 and PIP5K2 have functions in growth and development in various Arabidopsis tissues (Mei *et al*., 2012; Ischebeck *et al*., 2013; Tejos *et al*., 2014; Marhava *et al*., 2020), in line with a pronounced polarised distribution of PIP_2_ and PIP5Ks in different cell types and several stages of development (Ischebeck *et al*., 2013; Tejos *et al*., 2014). As a consequence, *pip5k1 pip5k2* double mutants exhibit severe defects in embryogenesis, vascular development and meristem formation in roots and shoots (Tejos *et al*., 2014; Marhava *et al*., 2020). PIP_2_ has also been implicated in protophloem differentiation (Rodriguez‐Villalon *et al*., 2015; Gujas *et al*., 2017; Marhava *et al*., 2020), where PIP5K1, PIP5K2 and PIP5K7 seem to be involved (Bauby *et al*., 2007; Marhava *et al*., 2020). Estradiol‐inducible overexpression of a human PIP5K in Arabidopsis dramatically increased PIP_2_ levels at the cost of PIP, and severely affected phloem and xylem differentiation (Gujas *et al*., 2017). Mutants in 5‐phosphatases (5‐PTases; i.e. CVP2, CVL1) that normally degrade PIP_2_ back into phosphatidylinositol 4‐phosphate (PI4P) also exhibit vascular differentiation defects (Rodriguez‐Villalon *et al*., 2015), and these genes were previously characterised for their role in cotyledon vascular patterning (Carland and Nelson, 2009). Independently, these findings highlight a role for PIP_2_ in development as well as stress signalling. Despite the obvious and prominent roles of PIP_2_, only little is known about how PIP_2_ production is triggered by upstream signalling pathways and how the lipid mediates its downstream effects.

In this work, we address the physiological function of PIP_2_ that is triggered in Arabidopsis roots by polyamines. Polyamines are small and versatile organic cations, present in all living organisms (Michael, 2016). The most common polyamines are putrescine (Put), spermidine (Spd) and spermine (Spm), representing a di‐, tri‐ and tetraamine, respectively. In plants, polyamines are implicated in stress responses, like drought, salinity and heat (Tiburcio *et al*., 2014; Michael, 2016). The molecular mechanisms behind polyamine perception and signalling are largely unknown, though several downstream components of polyamine action have been identified, including protein kinases, transcription factors, reactive oxygen species (ROS) and Ca^2+^ and K^+^ fluxes (Takahashi *et al*., 2003; Yoda, 2006; Kusano *et al*., 2008b; Wu *et al*., 2010; Bitrián *et al*., 2012; Moschou *et al*., 2012; Pál *et al*., 2015; Sagor *et al*., 2015; Pegg, 2016; Zarza *et al*., 2019). Polyamines have also recently been linked to the perception of salt stress through SOS1 (Chai *et al*., 2020). As polyamines appear to be involved in a range of plant stresses that also trigger PIP_2_ responses (Tiburcio *et al*., 2014; Heilmann, 2016a) and affect similar targets, including K^+^ channels (Liu *et al*., 2005; Ma *et al*., 2009; Wigoda *et al*., 2010), we hypothesised that polyamines are linked to the metabolism of phosphoinositides. This notion was supported by earlier studies, reporting effects of polyamine treatment on signalling phospholipids in spinach (*Spinacia oleracea*) hypocotyls (Dureja‐Munjal *et al*., 1992) and coffee (*Coffea arabica*) cells (Echevarría‐Machado *et al*., 2005).

Here, we show that polyamines, in particular Spm, trigger a rapid increase in PIP_2_ in the plasma membrane of Arabidopsis root cells. We further provide biochemical and genetic evidence that PIP_2_ is generated through activation of PIP5K7 and PIP5K9, and has a physiological role in regulating K^+^ fluxes.

## RESULTS

### Polyamines trigger rapid PIP_2_ responses in Arabidopsis roots

**Figure 1:**
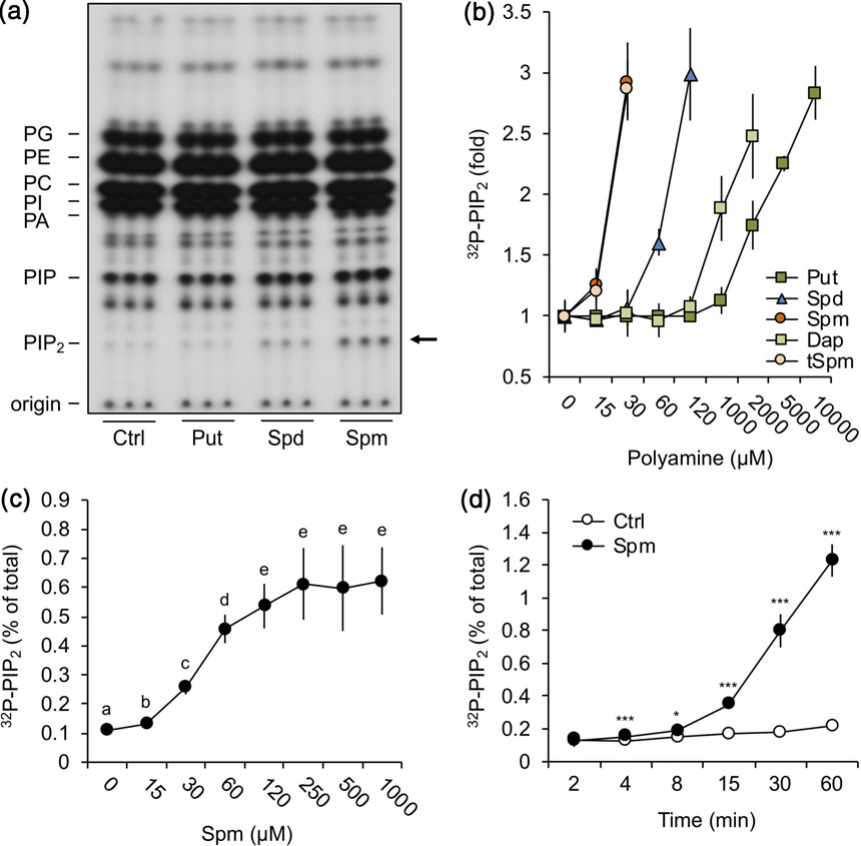
Polyamines trigger the formation of PIP_2_ in Arabidopsis seedlings. ^32^P_i_‐pre‐labelled seedlings were treated for 30 min with 60 µm of putrescine (Put), spermidine (Spd) or spermine (Spm), or with buffer alone (control, Ctrl), after which their lipids were extracted, separated by thin‐layer chromatography and visualised by autoradiography. (a) Autoradiograph of typical TLC, containing three samples per treatment. Abbreviations: PA, phosphatidic acid; PC, phosphatidylcholine; PE, phosphatidylethanolamine; PG, phosphatidylglycerol; PI, phosphatidylinositol; PIP, phosphatidylinositol phosphate; PIP_2_, phosphatidylinositol 4,5‐bisphosphate. (b) Quantified ^32^P‐PIP_2_ response after 30 min treatment with Put, Spd, Spm, diaminopropane (Dap) or thermospermine (tSpm) at the indicated concentrations, calculated as fold increase compared with control. Data are presented as the mean ± SD (*n* = 6). (c) Dose–response with Spm for 30 min and (d) time‐course with 60 µm Spm or buffer alone, showing the percentage of ^32^P‐PIP_2_ with respect to the total of ^32^P‐labelled phospholipids. In all cases, data are presented as the mean ± SD (*n* = 6).

The effect of polyamines on the formation of membrane phospholipids was analysed by *in vivo* radiolabelling using intact Arabidopsis seedlings, which were pre‐labelled overnight with ^32^P_i_ and treated the next day for 30 min with physiological concentrations (60 µm) of Put, Spd or Spm (Tassoni *et al*., 2000). As shown in Figure 1a, a substantial increase in PIP_2_ was found with Spd and Spm, but not with Put, which only induced a PIP_2_ response at much higher (mm) concentrations (Figure 1b). In contrast, Spm already induced a PIP_2_ response at 15 µm, whereas for Spd, concentrations of ≥60 µm were required. (Figure 1a,b). Thermospermine (tSpm), an isomer of Spm, was equally potent as Spm (Figure 1b), while the diamine diaminopropane (Dap) behaved similarly to Put (Figure 1b). These results indicate that the capacity of polyamines to trigger PIP_2_ is a function of the number of amine groups (hence, positive charge) rather than their molecular structure, with Spm^4+^ = tSpm^4+^ > Spd^3+^ >> Put^2+^ ≈ Dap^2+^.

So far, only hyperosmotic stress, heat and wounding have been shown to trigger PIP_2_ responses (Pical *et al*., 1999; DeWald *et al*., 2001; Heilmann *et al*., 2001; van Leeuwen *et al*., 2007; König *et al*., 2007, 2008; Mosblech *et al*., 2008; Mishkind *et al*., 2009). To explore the effect of polyamines in more detail, Spm was used, being the most potent compound. As shown in Figure 1c, a dose‐dependent PIP_2_ increase was observed when seedlings were treated for 30 min with different concentrations of Spm. PIP_2_ was already detectable at low µm concentrations and reached a maximum of ~4.5‐fold increase with 60 µm Spm. Time‐course experiments using 60 µm Spm revealed a rapid PIP_2_ increase, starting within 4 min after treatment and continuing exponentially until at least 60 min (Figure 1d).

As Spm can be catabolised by polyamine oxidases, which are known to cause H_2_O_2_ and NO accumulation (Tun, 2006; Moschou *et al*., 2008a; Zarza *et al*., 2019), we investigated whether Spm itself or one of its downstream oxidation products induced the PIP_2_ response using the ROS and NO scavengers DMTU and cPTIO, respectively. As shown in Figure S1, neither scavenger affected the Spm‐induced PIP_2_ response, while the accumulation of H_2_O_2_ and NO was reduced (Zarza *et al*., 2019). These results suggest that Spm itself rather than its metabolites triggered the formation of PIP_2_.

Earlier, we discovered that Spm triggered a rapid increase in phosphatidic acid (PA) levels, generated through phospholipase Dδ (PLDδ; Zarza *et al*., 2019). Determining both lipid responses in time‐course and dose–response experiments revealed a coordinated pattern (Figure S2a,b), which may reflect an interdependency since PLDδ has been suggested to be regulated by PIP_2_ (Li *et al*., 2009; Hong *et al*., 2016).

### Spm‐induced PIP_2_ is triggered by PIP5K activation

**Figure 2:**
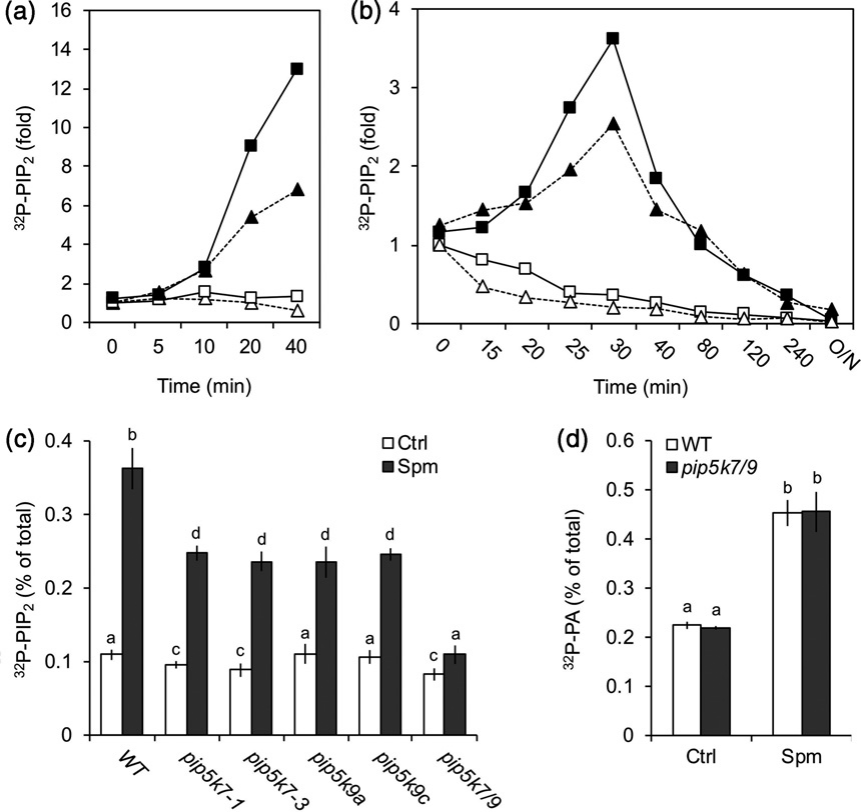
Spm‐induced PIP_2_ is triggered by activation of PIP5K, not through inhibition of PLC or PIP_2_ phosphatase. (a) Seedlings were pulse‐labelled with ^32^P_i_ for 15 min and then treated with Spm or buffer alone (Ctrl) for the times indicated. The PIP_2_ fold increase of two independent experiments is shown (squares and triangles). (b) Pulse‐chase experiment where seedlings were treated with buffer ± Spm for 15 or 30 min (triangles and squares, respectively), labelled with ^32^P_i_ for 5 min and then chased (*t* = 0) with non‐radioactive P_i_ in the presence or absence of Spm for the times indicated. Values in (a) and (b) were normalised to ^32^P‐PI and expressed with respect to Ctrl, 0 min. Open symbols, Ctrl; closed symbols, Spm. (c) ^32^P‐PIP_2_ response to Spm of WT and *pip5k7*, *pip5k9* and *pip5k7 pip5k9 (pip5k7/9)* double mutants. Seedlings were ^32^P_i_‐labelled overnight and treated for 30 min with 60 μm Spm. (d) ^32^P‐PA response in WT and *pip5k7/9* mutant plans. Data show that the PA response is independent of PIP_2_. In all cases, data are presented as the mean ± SD (*n* = 3).

Theoretically, the accumulation of PIP_2_ could result from three enzymatic routes: (i) through stimulation of its synthesis by PIP5K, (ii) through inhibition of its breakdown by PLC or (iii) through inhibition of its breakdown by PIP_2_ phosphatase. To distinguish between these possibilities, a differential ^32^P_i_‐labelling protocol was carried out that enhances the effect of kinase activity (Munnik *et al*., 1998b; Arisz and Munnik, 2013). The experimental approach exploits the fact that when ^32^P_i_ is added to seedlings, radiolabelled phosphate is rapidly incorporated into ATP, and hence into lipids that are phosphorylated in an ATP‐dependent fashion by kinases (i.e. PIP_2_ via PIP5K). When seedlings were short‐time labelled for 15 min and then treated with Spm, a massive increase in ^32^P‐PIP_2_ was observed, indicating that PIP_2_ was formed via PIP5K activation (Figure 2a). Similarly, pulse‐chase experiments in which seedlings were first treated with Spm, then labelled for 5 min with ^32^P_i_ and subsequently chased with non‐radioactive P_i_ revealed a quick rise in ^32^P‐PIP_2_ after stimulation, which then decreased again due to the rapid incorporation of non‐radioactive P_i_ (Figure 2b). This pattern is consistent with the notion that the Spm‐triggered PIP_2_ increase is a result of enhanced PIP5K activity, and not of inhibited PIP_2_ breakdown by PLC or 5‐PTase, because in those cases PIP_2_ levels would have remained high.

### Spm‐induced PIP_2_ is generated by PIP5K7 and PIP5K9

The Arabidopsis genome encodes 11 PIP5Ks (Mueller‐Roeber and Pical, 2002). To identify potential PIP5Ks involved, transfer DNA (T‐DNA) insertion mutants were tested for their ^32^P‐PIP_2_ response after Spm treatment (Figure S3a). A substantially reduced PIP_2_ response was found for T‐DNA mutants in *PIP5K7* or *PIP5K9* (Figure 2c). Individual knockout (KO) alleles of *pip5k9a*, *pip5k9c* or *pip5k7‐1*, as well as the knockdown line *pip5k7‐3* (Figure S3b,c), all showed a ~45% reduction in Spm‐induced PIP_2_, whereas a *pip5k7‐1 pip5k9c* double KO mutant (in figures abbreviated as *pip5k7/9*) typically lost ≥90% of its PIP_2_ response compared to wild type (WT) plants (Figure 2c). These results again confirm that the increase of PIP_2_ in response to polyamines is generated through PIP5K, and PIP5K7 and PIP5K9 are identified as the main isoforms involved. Despite suppressed PIP_2_ responses, the *pip5k* mutants exhibited WT PA responses (Figure 2d). Similarly, we found that the *pldδ* mutant revealed normal PIP_2_ responses upon Spm treatment (Figure S4). Together, these observations have three important implications, namely (i) the Spm‐induced PIP_2_ response does not reflect PA via the PLC/DGK pathway, in agreement with the finding that PA is predominantly generated by PLDδ (Zarza *et al*., 2019); (ii) the increase in PIP_2_ does not function as an activator of PLDδ, a notion based on *in vitro* findings and suggested to function *in vivo* (Pappan *et al*., 1997; Qin *et al*., 1997; Munnik and Testerink, 2009); (iii) PIP_2_ is likely involved in signalling by itself.

### *PIP5K7* and *PIP5K9* expression and regulation by Spm

**Figure 3:**
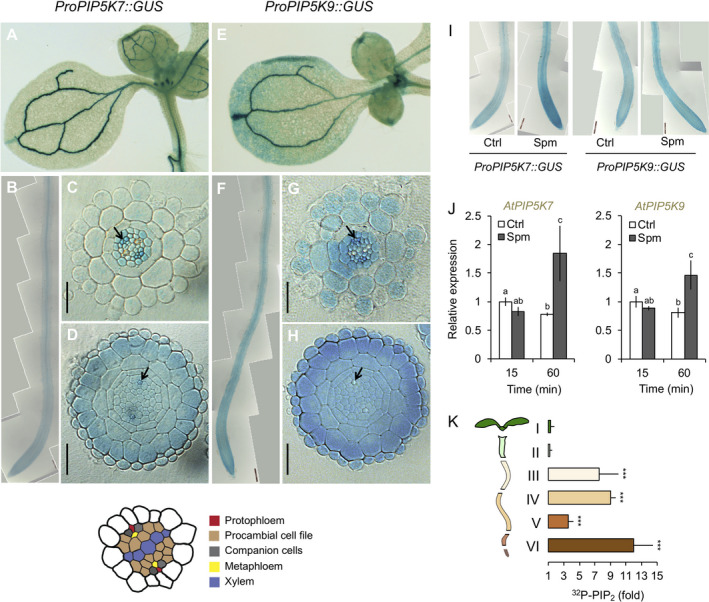
Expression of *PIP5K7* and *PIP5K9* in Arabidopsis seedlings. Histological GUS analyses of 5‐day‐old transgenic lines expressing (a–d) *ProPIP5K7::GUS* or (e–h) *ProPIP5K9::GUS*. Pictures show (a, e) cotyledons, (b, f) a general overview of the root and cross‐sections of (c, g) the root differentiation zone and (d, h) the division zone (root meristem). In (c, d, g, h), black arrowheads mark the protophloem cells. Results were confirmed in three independent transgenic lines. Bars represent 25 µm. (i, j) Effects of Spm on the expression of *PIP5K7* and *PIP5K9* genes using (i) a GUS reporter system and (j) RT‐PCR. (k) ^32^P‐PIP_2_ response in different sections of Arabidopsis seedlings. Type of section/length: (I) root tip, 2 mm, (II) root tip, 3 mm, (III) root tip, 5 mm, (IV) root tip, 5–7 mm, (V) hypocotyl and (VI) cotyledons. Results are expressed as the fold increase of PIP_2_ with respect to control treatment of each section. In all cases, data are presented as the mean ± SD (*n* = 3).

To test expression patterns and transcriptional activation of the *PIP5K7* and *PIP5K9* genes by Spm, transgenic Arabidopsis lines expressing promoter–GUS fusions of *PIP5K7* or *PIP5K9* were generated and GUS activity was visualised using histochemistry (Figure 3). In untreated seedlings, both *ProPIP5K7::GUS* and *ProPIP5K9::GUS* reporters revealed predominant promoter activity in the vasculature of cotyledons (Figure 3a,e) and roots (Figure 3b,f) and in most cells of the root meristem (Figure 3d,h), which was consistent with an earlier promoter–reporter gene fusion of *PIP5K7* (Bauby *et al*., 2007). In root elongation and differentiation zones (Figure 3b,f), *PIP5K7* expression appeared to be restricted to the stele, pericycle and phloem, with companion cells showing the highest expression levels together with metaphloem and procambium (Figure 3c). By contrast, *PIP5K9* seemed to be expressed in all cells of the elongation and differentiation zones, except in the endodermis, where its expression was limited to cells adjacent to the xylem pole pericycle (Figure 3g). Interestingly, discontinuous expression of *PIP5K9* was detected in the epidermal cell layer (Figure 3g).

Spm strongly induced GUS staining of the *ProPIP5K7::GUS* reporter in the root tip meristem and stele, whereas only a slight increase in the activity of the *PIP5K9* promoter was observed after 30 min (Figure 3i). Quantitative real‐time PCR (qRT‐PCR) analyses confirmed the results from the promoter–GUS fusions, where between 15 and 60 min after Spm treatment an increased transcript abundance was detected for both genes (Figure 3j). These results indicate that enhanced *PIP5K7*/*PIP5K9* expression may also contribute to the increased PIP_2_ formation upon Spm treatment.

To correlate *PIP5K* expression and PIP_2_ formation in more detail, ^32^P_i_‐labelling and Spm treatment were performed as before, but now tissues were dissected after sample fixation, and the PIP_2_ responses were determined in the different sections (Figure 3k). Interestingly, Spm only triggered a PIP_2_ response in the root, not in the shoot or hypocotyl, in contrast to the clear vascular expression of both genes (Figure 3a,e). Within the root, the strongest PIP_2_ responses were found near the tip and maturation zone (sections III, IV, VI; Figure 3k), which correlated well with the *PIP5K7* and *PIP5K9* expression in these tissues (Figure 3i). Repeating the experiment with higher Spm concentrations gave similar results (Figure S5a). This lack of responsiveness in hypocotyl and cotyledons could be a consequence of tissue‐specific Spm perception and/or transport, or the absence of auxiliary signalling elements required for post‐transcriptional PIP5K activation. We did observe PIP_2_ responses in isolated leaf discs of mature (3‐week‐old) plants (Figure S5b).

### Phenotypic characterisation of *pip5k7 pip5k9* double mutants

No obvious growth or developmental phenotypes were observed in *pip5k7 pip5k9* double mutants, on either agar plates or soil (Figure S6a,b), which is in strong contrast to the severe developmental defects reported for *pipk5k1 pip5k2* (Ischebeck *et al*., 2013; Tejos *et al*., 2014; Marhava *et al*., 2020). The phenotypic differences between the *pip5k7 pip5k9* and *pip5k1 pip5k2* double mutants are likely due to local and/or temporal versus sustained changes in PIP_2_ concentrations, since the basal levels of PIP_2_ in *pip5k7 pip5k9* seedlings were only marginally reduced (Figure 2), while those in *pipk5k1 pip5k2* mutants were even very similar to WT (Figure S3a). Since *PIP5K7* and *PIP5K9* were strongly expressed in the stele, a more detailed analysis of the vascular tissue was performed. While no effect on phloem or xylem differentiation was found (Figure S6c), we did observe defects in the continuity of the veins of the cotyledons. While 2.4% of the WT seedlings contained discontinuous veins, for *pip5k7 pip5k9* mutants this increased up to 20%, which seemed to be mainly caused by *pip5k9* (24%), with only 10.9% discontinuous veins for *pip5k7* (Figure S6d). Overall, the data suggest that the PIP_2_ formation by PIP5K7 and/or PIP5K9 has only minor roles in basal plant development, but is dynamically induced in response to polyamines.

### Spm triggers the PIP_2_ response at the plasma membrane

To better understand the dynamic changes of PIP_2_ upon polyamine treatment, the subcellular localisation of the response *in vivo* was analysed using a fluorescent PIP_2_ biosensor (*ProUBQ10::YFP‐PH_PLCδ1_*) (Van Leeuwen *et al.,* 2007; Simon *et al*., 2014; Tejos *et al*., 2014). Confocal imaging was focused on the cortex cells in the transition zone of the root tip because these cells exhibit sufficient contrast of the biosensor signal between the plasma membrane and cytosol and because PIP_2_ responses are particularly high in that zone (Figure 3i).

**Figure 4:**
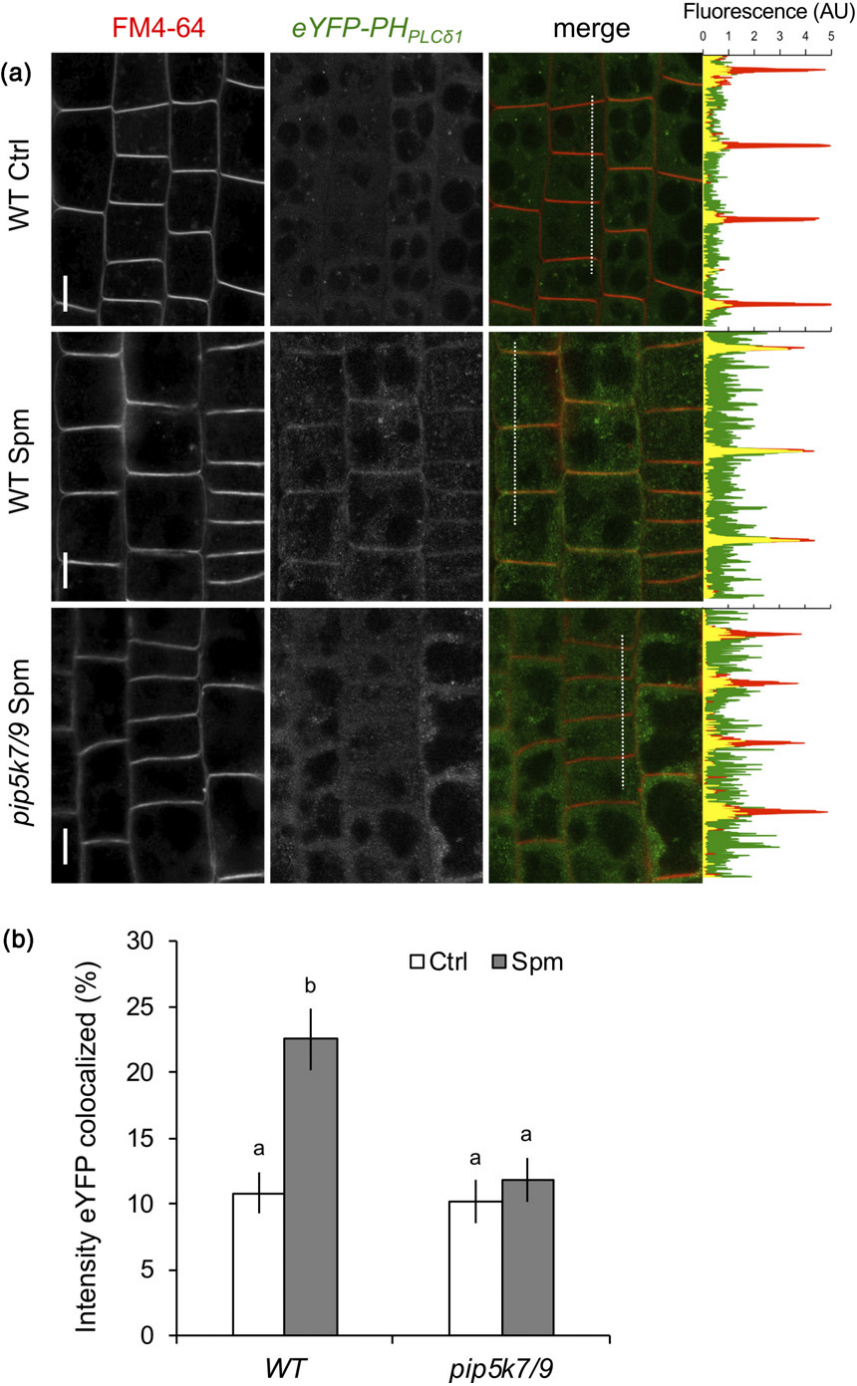
Spm‐induced PIP_2_ is generated at the plasma membrane. (a) Confocal images of WT and *pip5k7/9* seedlings expressing the PIP_2_ biosensor *ProUBQ10::eYFP‐PH_PLCδ1_* treated with buffer ± Spm at 30 min. Images show cortex cells in the root transition zone (left) and a representative plot‐profile analysis (right) indicating differences in membrane association (FM4‐64, red) of eYFP‐PH_PLCδ1_ (green). Units are expressed as arbitrary units (AUs). Bars represent 10 µm. (b) To quantify the colocalisation of eYFP signal with FM4‐64, the entire cell (including plasma membrane) was selected as the region of interest and the percentage of fluorescence intensity colocalisation was determined. Data are presented as the mean ± SD (*n* = 20).

At control conditions, most of the biosensor was located in the cytosol (Figure 4a) due to the low basal concentrations of PIP_2_ in the membrane (van Leeuwen *et al*., 2007; Vermeer and Munnik, 2013). However, in response to Spm, the biosensor was clearly recruited to the plasma membrane, resulting in pronounced colabelling with FM4‐64 (Figure 4a). By contrast, this response was strongly reduced in the *pip5k7 pip5k9* mutant (Figure 4b), confirming the involvement of PIP5K7 and PIP5K9 in the Spm‐induced PIP_2_ increase at the plasma membrane.

### Spm‐induced K^+^ efflux requires PIP_2_ production by PIP5K7 and PIP5K9

**Figure 5:**
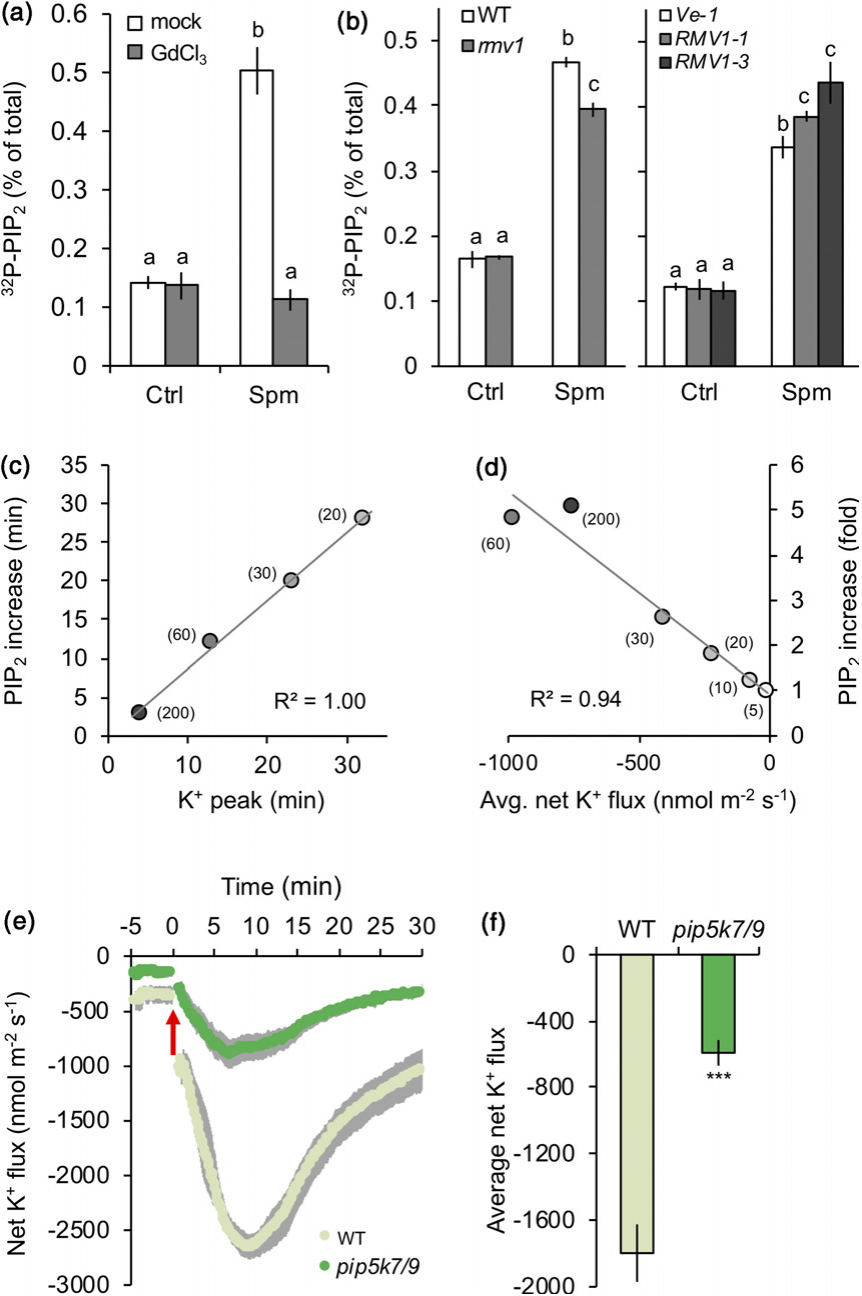
Spm‐induced K^+^ efflux relies on the formation of PIP_2_ and Spm transport across the plasma membrane. (a) The Spm‐induced PIP_2_ response is gadolinium (GdCl_3_)‐sensitive. Pre‐treatment with gadolinium, a well‐known polyamine uptake inhibitor, completely blocks the Spm‐induced PIP_2_ response. ^32^P‐pre‐labelled WT seedlings were pre‐treated for 60 min with 100 µm GdCl_3_, washed and then treated with or without 60 µm Spm for 30 min. (b) The polyamine uptake transporter RMV1 is required for a full PIP_2_ response. WT, *rmv1* and two *RMV1‐OE* lines were ^32^P‐labelled and tested for their PIP_2_ response in the absence or presence of 60 µm Spm for 30 min. For both cases, data are presented as the mean ± SD (*n* = 4). (c) Correlation between K^+^ efflux peak time and the PIP_2_ response at different Spm concentrations. (d) Correlation between net K^+^ efflux and the PIP_2_ response using different Spm concentrations (*t* = 30 min). (e) MIFE K^+^ flux kinetics in WT and *pip5k7/9* seedlings when 60 µm Spm was added (red arrow). (f) MIFE average K^+^ flux in WT and *pip5k7/9* mutant plants upon 60 µm Spm treatment. For all MIFE data, data are shown as the mean ± SE (*n* = 6–7); negative values represent *net efflux* of ions across the plasma membrane into the apoplast.

Due to their positive charge at physiological and acidic pH, polyamines are known to trigger a depolarisation of the plasma membrane, with a consequent efflux of K^+^ observed in several plant roots, including pea (*Pisum sativum*), maize (*Zea mays*) and Arabidopsis (Pandolfi *et al*., 2010; Zarza *et al*., 2019). In mammalian cells, almost all K^+^ channels are regulated by PIP_2_ (Dickson and Hille, 2019), and also in plants there are indications that it can regulate K^+^ transport (Liu *et al*., 2005; Ma *et al*., 2009; Wigoda *et al*., 2010). To investigate a potential link between polyamine‐triggered PIP_2_ and K^+^ flux, we used gadolinium (GdCl_3_), which is an inhibitor of Spm uptake across the plasma membrane (Pistocchi *et al*., 1988; Ditomaso *et al*., 1992b; Pottosin and Shabala, 2014). Pre‐treatment of Arabidopsis seedlings with gadolinium blocked the Spm‐induced K^+^ efflux (Zarza *et al*., 2019), and inhibited the Spm‐induced PIP_2_ response (Figure 5a). This indicates that the effect of exogenous Spm on K^+^ currents and PIP_2_ may be triggered from the cytosolic side of internalised Spm, consistent with other reports (e.g. Liu *et al*., 2000). Part of the Spm appears to be transported via resistant to methyl viologen 1 (RMV1), a plasma membrane‐localised Spm uptake transporter (Fujita *et al*., 2012), since reduced PIP_2_ responses were observed in Arabidopsis *rmv1* mutants and increased PIP_2_ responses when *RMV1* was overexpressed (Figure 5b). These results link polyamine uptake and PIP_2_ responses.

As polyamines influenced both changes in PIP_2_ and K^+^ flux, we next tested whether *pip5k7 pip5k9* mutants were affected in K^+^ transport. Using non‐invasive microelectrode ion flux measurement (MIFE), Spm was found to induce a dose‐ and time‐dependent efflux of K^+^ in WT plants (Zarza *et al*., 2019). Comparing the loss of K^+^ with the onset of a significant PIP_2_ accumulation in time (Figure 5c) or with the intensity of the response (fold increase) after 30 min (Figure 5d), strong correlations were observed (*R* ^2^ = 1.00 and 0.94, respectively). Importantly, ~70% of the Spm‐triggered K^+^ efflux response was lost in *pip5k7 pip5k9* mutants compared to WT (Figure 5e,f). Together, these results indicate (i) that Spm must be taken up into the cell to exert its function; (ii) that PIP_2_ is an element of the signalling cascade that links Spm with the efflux of K^+^ at the plasma membrane; and (iii) that an Spm‐induced increase of PIP_2_ at the plasma membrane functions upstream of inducing this K^+^ efflux.

### *Pip5k7 pip5k9* double mutants display enhanced sensitivity to Spm or KCl

**Figure 6:**
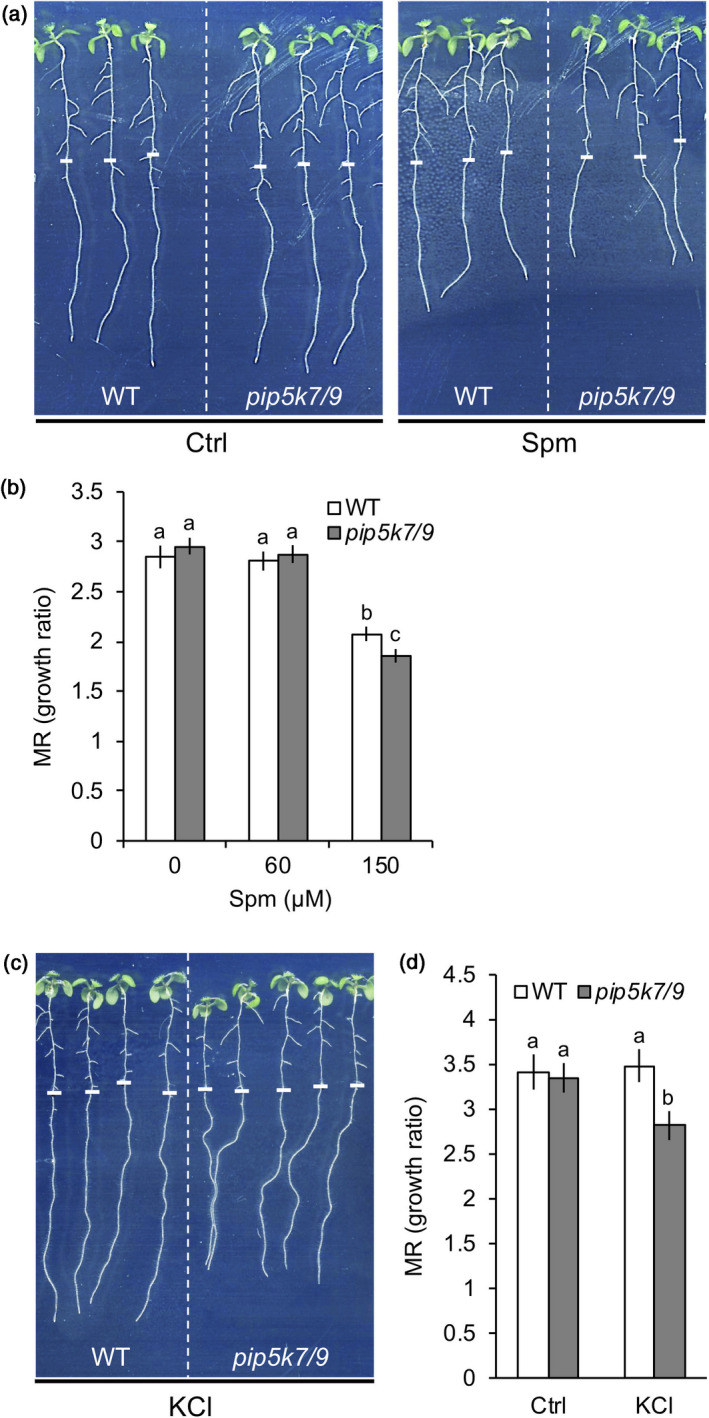
Effects of Spm and KCl on seedling root growth in WT and *pip5k7/9* mutant plants. Five‐day‐old seedlings were transferred to plates supplemented with (a, b) Spm or (c, d) KCl and grown for 4 more days. (a) Phenotype of WT and mutant seedlings grown with and without 150 µm Spm. (b) Effects of Spm on main root (MR) growth of WT and *pip5k7/9* seedlings. (c, d) Phenotype of WT and mutant seedlings grown with and without of 50 mm KCl. Results are expressed as the MR growth ratio. Data are presented as the mean ± SD (*n* = 40). White dashes indicate the position of the root tip when seedlings were transferred.

Since the application of polyamines has previously been shown to affect root growth in seedlings (Couée *et al*., 2004; Jancewicz *et al*., 2016; Zarza *et al*., 2019), the effect in *pip5k7 pip5k9* mutants was analysed. Five‐day‐old WT and *pip5k7 pip5k9* double mutant seedlings were transferred to agar plates containing increasing concentrations of polyamines, and after 4 days, a substantially higher degree of root growth inhibition was observed for the double mutants compared to WT (Figure 6a,b). Due to the high potassium permeability of the plasma membrane (resulting from the presence of membrane channels), the cytoplasmic potassium concentration is very sensitive to changes in the membrane potential (Maathuis and Sanders, 1997). Conversely, when the apoplastic potassium level increases, a large influx of this cation will cause a reduction in the membrane potential, which, if not corrected, can cause deleterious effects on other transporters driven by the membrane potential (Haruta and Sussman, 2012). To test whether the increased sensitivity was related to the decreased capacity of the mutant to maintain K^+^ homeostasis, we increased the external K^+^ levels by adding 50 mm KCl to the agar plates. As shown in Figures 6c,d, while WT seedlings showed no apparent phenotype, and *pip5k7 pip5k9* mutants showed a hypersensitive response revealed by a clear reduction in root growth, which is a typical response to higher KCl concentrations (Haruta and Sussman, 2012). These results further support a link between PIP_2_ production by PIP5K7 and PIP5K9 and the regulation of K^+^ transport.

## DISCUSSION

Polyamines are naturally occurring polycationic molecules involved in multiple processes along a cell’s lifespan (Tiburcio *et al*., 2014; Miller‐Fleming *et al*., 2015). The elucidation of their mode of action to understand their pleiotropic effects has become a major challenge in biology. Here, we obtained biochemical and genetic evidence (i) that polyamines trigger a rapid PIP_2_ response in the plasma membrane of root cells through activation of PIP5K7 and PIP5K9 and (ii) that the increase of PIP_2_ modulates the flux of K^+^ across the plasma membrane. This polyamine‐induced PIP_2_ response was time‐, dose‐ and charge‐dependent, where the effects were ordered as follows: Spm^4+^ = t‐Spm^4+^ > Spd^3+^ > Put^2+^ = Dap^2+^ (Figures 1 and S1). Together with earlier observations in spinach hypocotyls (Dureja‐Munjal *et al*., 1992) and coffee cells (Echevarría‐Machado *et al*., 2005) and ample evidence in animal systems (Coburn, 2009), these results indicate the effect of polyamines on phosphoinositide signalling may have been conserved across kingdoms.

Polyamines are secreted in response to environmental cues such as drought and salt stress and in response to abscisic acid (Moschou *et al*., 2008b; Toumi *et al*., 2010). Once in the apoplast, they can be metabolised by polyamine oxidases, generating ROS and triggering downstream effects (Takahashi *et al*., 2003; Moschou *et al*., 2008a; Toumi *et al*., 2010; Pottosin and Shabala, 2014). However, their transport across the plasma membrane of cells distal from where the polyamines were secreted represents another important event (Ditomaso *et al*., 1992a; Angelini *et al*., 2010; Campestre *et al*., 2011; Moschou *et al*., 2012). The uptake of polyamines is very rapid, reaching saturation at µm concentrations and causing intracellular polyamine concentrations to rise by 10–1000 µm min^−1^ (Pistocchi *et al*., 1987, 1988; Ditomaso *et al*., 1992a; Echevarría‐Machado *et al*., 2005). In that regard, several polyamine uptake transporters (PUTs) have been recently identified, including RMV1/PUT3, which transports Spm across the plasma membrane (Fujita *et al*., 2012; Martinis *et al*., 2016). Our data indicate that RMV1/PUT3 is involved in the Spm uptake route that triggers PIP_2_ (Figure 5). This pathway is likely redundant as there are several members of the PUT and amino acid‐polyamine‐choline transporter family that could transport polyamines (Verrey *et al*., 2004; Rentsch *et al*., 2007; Mulangi *et al*., 2012; Fujita and Shinozaki, 2014), as well as other types, including the nitrogen transporter family (Tong *et al*., 2016).

Importantly, Spm concentrations found to be effective in this work (low µm range) are significantly lower than the basal polyamine levels in plant cells (Galston and Kaur‐Sawhney, 1995), and much lower than concentrations frequently used in other studies, where typically high µm/low mm concentrations have been used (Sagor *et al*., 2015; Marco *et al*., 2019; Tajti *et al*., 2019). In our hands, the effective concentration of Spm to induce PIP_2_ revealed a clear saturation response at low µm concentrations (Figure 1) and correlated with the dose‐dependent inhibition of root growth (Figure 6; Zarza *et al*., 2019). Interestingly, PIP_2_ hyperaccumulation and root growth inhibition are also observed in the Arabidopsis *sac9* mutant, a 5‐PTase involved in PIP_2_ degradation (Williams *et al*., 2005), and in seedlings where PIP_2_ synthesis was boosted by overexpression of a human *PIP5K* (Im *et al*., 2014; Gujas *et al*., 2017).

In contrast to animal systems, phosphorylation of PI4P at the 5‐position of the inositol ring is the only route to synthesise PIP_2_ in plants (Munnik and Testerink, 2009, Munnik and Vermeer, 2010; Heilmann, 2016a). Radiolabelling and pulse‐chase experiments (Figures 1 and 2) indicated that the increase in PIP_2_ upon polyamine treatment was caused by PIP5K activation, and this was confirmed by a complementary reverse‐genetics approach, which identified PIP5K7 and PIP5K9 as the main enzymes involved. Expression of the *PIP5K7* and *PIP5K9* genes was also induced upon polyamine treatment (Figure 3); however, the rapid formation of PIP_2_ within minutes is more in favour of a post‐translational mode of activation, for which the mechanism is still unknown. PIP5K activity can be regulated by protein phosphorylation (Westergren *et al*., 2001; Hempel *et al*., 2017; Menzel *et al*., 2019). Checking the PhosPhAt database (Durek *et al*., 2010) revealed that PIP5K7 could be phosphorylated on serine‐224, but information on PIP5K9 is lacking. In principle, polyamines could increase PIP5K activity by interacting directly with the kinase and ATP‐Mg^2+^ (Meksuriyen *et al*., 1998). *In vitro*, human PIP5K can be activated by Spm and Spd (Bazenet *et al*., 1990; Singh *et al*., 1995; Chen *et al*., 1998), but whether this is also true for plant enzymes is unknown. Why this would be specific for PIP5K7 and PIP5K9 and leave other PIP5Ks expressed in the same tissues (e.g. PIP5K1–4) unaffected is also unknown. Indirect effects of polyamines could occur, for example, by modulating protein effectors such as 14‐3‐3 proteins (Athwal and Huber, 2002; Garufi *et al*., 2007) or stimulating Ca^2+^ influx (Takahashi *et al*., 2003). In animal cells polyamines can also regulate the activity of Rho‐kinase (Rao *et al*., 2003), which can stimulate PIP5K activity too (Oude Weernink *et al*., 2000). Plant PIP5Ks may be regulated differently, however, as indicated by the multiple Membrane Occupation and Recognition Nexus (MORN) motifs that are present in PIP5K1–9 from Arabidopsis, but lacking in animal and yeast PIP5Ks (Ma *et al*., 2006). Part of the increase in PIP5K activity may additionally be explained by the upregulation of *PIP5K7* and *PIP5K9* expression after prolonged Spm stimulation (Figure 3i,j). However, the rapid and specific activation of PIP5K7 and PIP5K9 is likely a more direct response to polyamines, possibly via a rapid post‐translational modification, as has been suggested previously for other plant PIP5Ks (Westergren *et al*., 2001; Heilmann and Heilmann, 2015; Heilmann, 2016b).

While *PIP5K7* and *PIP5K9* appear to be expressed in both shoot and root vasculature (Figure 3), the Spm‐induced PIP_2_ response was only observed in the root, with the highest activity near the tip (Figure 3k). This pattern suggests differences in perception and/or transport of Spm, or in the signalling pathway linking polyamines with the PIP_2_ increase in these tissues. Interestingly, the endogenous synthesis of Spm by spermine synthase (SPMS) and of tSpm by ACAULIS5 (ACL5) is also restricted to the vasculature, that is, phloem and xylem, respectively (Vera‐Sirera *et al*., 2015; Zarza *et al*., 2019). Indeed, plants with increased endogenous levels of Spm by overexpressing SPMS (Sagor *et al*., 2013) contained significantly higher basal amounts of PIP_2_ (Figure S7), supporting the idea of an intrinsic SPMS/PIP5K module that controls developmental aspects of root formation or root responses to environmental stresses. This is also supported by the fact that the Arabidopsis *acl5* mutant is heavily perturbed in xylem differentiation (Muñiz *et al*., 2008) and that overexpression of human *PIP5K* in Arabidopsis seedlings caused severe defects in phloem and xylem differentiation (Gujas *et al*., 2017). Regulation of protophloem differentiation and vascular development is also known to involve phosphoinositides (Carland and Nelson, 2004; Carland and Nelson, 2009; Rodriguez‐Villalon *et al*., 2015; Gujas *et al*., 2017; Marhava *et al*., 2020; Figure S6).

While *pip5k7 pip5k9* mutants displayed subtle defects in vein continuity in cotyledons, no macroscopic plant phenotypes were observed (Figure S6), which is in huge contrast to the dramatic root and developmental phenotypes found in *pip5k1 pip5k2* mutants (Ischebeck *et al*., 2013; Tejos *et al*., 2014), root hair phenotypes found in *pip5k3* and *pip5k4* (Stenzel *et al*., 2008; Kusano *et al*., 2008a; Wada *et al*., 2015) and developmental disorders found in pollen tubes in *pip5k4–6* and *pip5k10–11* mutants (Sousa *et al*., 2008; Ischebeck *et al*., 2008; Zhao *et al*., 2010; Ischebeck *et al*., 2011). The fact that flowering plants have so many specific PIP5Ks and PIP_2_‐dependent cellular responses despite their low PIP_2_ levels is fascinating in itself. Evidently, PIP_2_ increases will be very transient and/or localised, and may be triggered by upstream signalling pathways according to particular cellular requirements. Advanced imaging of Arabidopsis cells of the root transition zone showed that Spm triggered the recruitment of the PIP_2_ biosensor to the plasma membrane, and that this recruitment was strongly reduced in the *pip5k7 pip5k9* double mutant (Figure 4). Salt and heat stress also induced the accumulation of PIP_2_ at the plasma membrane (van Leeuwen *et al*., 2007; König *et al*., 2008; Mishkind *et al*., 2009; Simon *et al*., 2014; Menzel *et al*., 2019), suggesting a potential link to polyamines in stress conditions at which polyamines have also been shown to increase.

So far, the function of these dynamic stress‐induced PIP_2_ responses at the plasma membrane remains unclear, even though a role for clathrin‐mediated endocytosis has been proposed (König *et al*., 2008; Zhao *et al*., 2010; Mei *et al*., 2012; Ischebeck *et al*., 2013; Tejos *et al*., 2014; Menzel *et al*., 2019). Studies on tip‐growing cells suggested that PIP_2_ does not freely diffuse from its site of production, but may be channelled toward specific downstream effectors by processes depending on the interaction of PIP5K with these targets (Saavedra *et al*., 2012; Stenzel *et al*., 2012; Heilmann and Heilmann, 2013; Tejos *et al*., 2014). This channelling hypothesis is consistent with the notion that PIP_2_ colocalises with PIP5Ks in pollen tubes (Sousa *et al*., 2008; Ischebeck *et al*., 2008; Zhao *et al*., 2010; Ischebeck *et al*., 2011; Stenzel *et al*., 2012; Ugalde *et al*., 2016), root hairs and certain root cells (Stenzel *et al*., 2008; Thole *et al*., 2008; Kusano *et al*., 2008a; Vermeer *et al*., 2009; Tejos *et al*., 2014). In that sense, it is tempting to speculate that the accumulation of PIP_2_ at the plasma membrane in response to polyamines results from plasma membrane‐localised PIP5K7 and PIP5K9. Transient expression of PIP5K9‐GFP in tobacco (*Nicotiana benthamiana*) mesophyll cells and onion epidermal cells revealed fluorescence at the plasma membrane and nucleus (Lou *et al*., 2007). However, we never observed nuclear accumulation of the PIP_2_ biosensor upon Spm treatment, while we did observe this after prolonged heat stress (Mishkind *et al*., 2009). Arabidopsis PIP5Ks can contain functional nuclear localisation sequences (Gerth *et al*., 2017b), so it is possible that dynamic re‐localisation of PIP5Ks between cytoplasm, plasma membrane, nucleus and possibly other subcellular locations may occur to meet specific cellular requirements. So far, the complexity of plant PIP_2_ distribution and its functions are just emerging, and even the data available must be reviewed with caution, because different model systems have been used, including heterologous expression in different plant species, transient expression in cultured cells and stable expression in transgenic Arabidopsis plants.

Polyamine uptake in plant roots cause the plasma membrane to depolarise (Ozawa *et al*., 2010; Pottosin *et al*., 2014), resulting in a net efflux of K^+^ (Pandolfi *et al*., 2010; Zepeda‐Jazo *et al*., 2011). Besides its important role as counter‐ion for keeping the membrane potential in the cellular compartments (Dreyer and Uozumi, 2011), K^+^ is also a major cation that osmotically steers various turgor‐driven processes (Maathuis, 2009). In that sense, both K^+^ and polyamines have been speculated to play similar physiological roles, acting as a mutual counter‐balance, for a long time (Richards and Coleman, 1952). Using MIFE, a strong and transient efflux of K^+^ from epidermal cells of the root elongation zone was found upon Spm induction (Figure 5e; Zarza *et al*., 2019). The imbalance of the homeostatic‐K^+^ control caused by the efflux of K^+^ may lead to multiple downstream effects due to potassium’s capacity to activate K^+^‐sensitive enzymes (Marschner, 1995; Wu *et al*., 2018), or to function as a signalling agent itself (Demidchik, 2014; Shabala, 2017). Importantly, the loss of K^+^ during 30 min Spm treatment strongly coincided in time and intensity with the PIP_2_ response (Figure 5c,d). A causal effect of PIP_2_ on K^+^ fluxes can also be deduced from the observation that *pip5k7 pip5k9* seedlings exhibited a lower basal K^+^ efflux than WT at control conditions before the application of Spm (Figure 5e). This difference became much more pronounced upon Spm treatment, where ~70% less K^+^ was excluded from *pip5k7 pip5k9* than from WT (Figure 5e,f). Mutant seedlings were also more sensitive to Spm and KCl on plate growth assays (Figure 6), supporting a role for PIP5K7 and PIP5K9 in K^+^ homeostasis and placing PIP_2_ upstream of K^+^ flux, and providing a link with the inhibitory effect of Spm on root growth. In agreement, the tetraamine‐depleted *acl5 spms* double mutant is hypersensitive to KCl (Yamaguchi *et al*., 2006), reinforcing the idea of a polyamine/PIP_2_ module. In animal cells, most K^+^ channels are regulated by PIP_2_ (Dickson and Hille, 2019), and there are some indications for plants as well (Liu *et al*., 2005; Ma *et al*., 2009; Wigoda *et al*., 2010); here, genetic evidence for such a model is provided. An important next step will be to identify the K^+^ channels involved and whether the effect of PIP_2_ is direct or indirect. For example, the clustering of the Arabidopsis K^+^ channel GORK, which parallels its gating activity (Eisenach *et al*., 2014), could be PIP_2_‐dependent, or the suppression of the inward rectifier KAT1 (Eisenach *et al*., 2014), by removing it from the plasma membrane.

Future research on how PIP5K7 and PIP5K9 are activated, together with advanced electrophysiology experiments on *pip5k*7 *pip5k9* mutants and polyamine transporters, should provide further knowledge on the molecular mechanisms that underlie these newly identified cellular and physiological responses.

## EXPERIMENTAL PROCEDURES

### Plant material and growth conditions

*Arabidopsis thaliana pip5k7‐1* (SALK_151429), *pip5k7‐3* (SALK_107796), *pip5k8‐2* (SALK_040022) and *pip5k9c* (SALK_013602) T‐DNA insertion mutants and plants with the transposon allele *pip5k9a* (SM_3_39157) were obtained from the Nottingham Arabidopsis Stock Centre. Arabidopsis* rmv1, pip5k1 pip5k2, pip5k3‐2, pip5k3‐4, pip5k4 pip5k5, pip5k6 and pip5k10 pip5k11* mutant null alleles and *Pro35S::RMV1, Pro35S::SPMS‐1, Pro35S::SPMS‐15* and *ProUBQ10::YFP‐PH_PLCδ1_* transgenic lines were described previously (Ischebeck *et al*., 2008; Kusano *et al*., 2008a; Zhao *et al*., 2010; Ischebeck *et al*., 2011; Fujita *et al*., 2012; Sagor *et al*., 2013; Ischebeck *et al*., 2013; Simon *et al*., 2014). The *pip5k7/9* double mutant was obtained by crossing *pip5k7‐1* with *pip5k9c*. The *pip5k7/9* line was, in turn, crossed into a *ProUBQ10:YFP‐PH_PLCδ1_* line for confocal studies. In most cases, *A. thaliana* ecotype Col‐0 was used as WT, except for the *rmv1* and *Pro35S::RMV1* lines, in which Ler ecotype and Col‐0 empty vector, Ve‐1, were used as WTs, respectively.

Seeds were surface‐sterilised using chlorine gas and sown under sterile conditions on square Petri dishes containing standard growth medium consisting of ½ MS medium with Gamborg B5 vitamins (pH 5.7; KOH), 1% (w/v) sucrose and 1% (w/v) agar. Plates were vernalised (4°C, 48 h) and placed vertically, under an angle of 70° in a growth chamber (16 h/8 h light/dark cycle, 110–130 µmol m^−2^ sec^−1^) at 22°C. Five‐day‐old seedlings were transferred to either 2 ml safe‐lock Eppendorf tubes for ^32^P_i_‐labelling experiments or to small round Petri dishes for incubations with polyamines and chemicals. Glass was avoided since polyamines tend to stick to glass surfaces. For gene expression analyses, seeds were germinated on agar plates containing a nylon mesh (Ø 43 μm) to facilitate the transfer of the seedlings. Mature plants were grown on soil, under the same light and temperature regime as above.

### Identification of *pip5k7*, *pip5k9* and *pip5k7/9* double mutants

Genotyping of *pip5k7‐1*, *pip5k7‐3*, *pip5k9a*, *pip5k9c* and *pip5k7‐1/pip5k9c* and isolation of homozygous mutant lines was performed by PCR using a combination of gene‐ and T‐DNA‐specific (SALK‐LB) primers (Table S1). For gene expression analyses, RNA from WT and mutants was extracted using TRIzol reagent (Invitrogen, Waltham, MA, USA) and treated with Turbo DNase (Ambion, Waltham, MA, USA). cDNA was synthesised using the RevertAid Synthesis Kit (Fermentas, Waltham, MA, USA), and transcript levels of *AtPIP5K7* and* AtPIP5K9* were determined by semi‐quantitative RT‐PCR using gene‐specific primers (Table S1) and the following PCR conditions: 95°C 5 min, followed by 40 cycles of 95°C 30 sec, 52°C 30 sec and 72°C 2 min and a final elongation step at 72°C 6 min. *SAND* (*AT2G28390*) was used as a reference gene since it was shown to be very consistently expressed under various conditions (Hong *et al*., 2010).

### Reporter constructs

Promoter–GUS fusion constructs were generated as described previously (Stenzel *et al*., 2008). In brief, the GUSPlus gene was amplified from pCAMBIA1305.1 (AF354045) using primers described in Table S1, and the PCR product was introduced as *Not*I‐*Sac*I fragment into pGreen0029, yielding pGreenGUSPlus. As promoters, 1500 bp genomic sequences upstream of coding sequences of *PIP5K7* or *PIP5K9* were amplified from BAC clones T19D16 and F8A24, respectively, using primers described in Table S1. PCR products were moved directionally as *Sal*I‐*Not*I fragments into pGreenGUSPlus, and resulting plasmids transformed were into *Agrobacterium tumefaciens* strain EHA105 for transformation into Arabidopsis.

### ^32^P_i_‐phospholipid labelling, extraction and analysis

Phospholipid responses were measured as described earlier (Munnik and Zarza, 2013). Briefly, three seedlings per sample were metabolically labelled overnight in 2 ml Eppendorf safe‐lock tubes containing 200 µl incubation buffer (2.5 mm MES‐KOH, pH 5.7, 1 mm KCl) and 2.5–10 µCi ^32^PO_4_^3−^ (1 µl stock ^32^P_i_; carrier‐free, 10 µCi µl^−1^; Perkin‐Elmer), in continuous light. For mature plants, leaf discs (∅ 5 mm) were taken from 3‐week‐old plants and labelled using the same conditions. Treatments started by adding 200 µl of a 2× solution and were stopped by adding 5% (v/v) perchloric acid. In general, treatments of 30 min and 60 µm Spm were used, unless indicated otherwise. Lipids were extracted and analysed by thin‐layer chromatography using an alkaline solvent (Munnik *et al*., 1994) or a water‐saturated ethyl acetate solvent system for PA analyses (Munnik and Laxalt, 2013). Radioactivity was visualised by autoradiography and quantified by phosphoimaging (Typhoon FLA 7000; GE Healthcare, Chicago, IL, USA).

For certain experiments, a modified ^32^P_i_‐labelling protocol was used: (i) for short‐labelling experiments, ^32^P_i_ was added 30 min prior to treatment; (ii) for pulse‐chase experiments, seedlings were first treated with Spm for 15 or 30 min and then labelled with ^32^P_i_ for 5 min, after which 1 mm P_i_ buffer (K_2_HPO_4_/KH_2_PO_4_, pH 5.7) was added in the presence or absence of Spm; (iii) for tissue dissection experiments, seedlings were treated and fixed as described above, after which they were carefully cut into sections with a scalpel and every section was extracted separately.

### GUS analysis

GUS staining was performed as described in Depuydt *et al*. (2013). Briefly, seedlings were incubated for 15 min in GUS staining buffer and mounted in chloral hydrate for immediate visualisation with a Leica compound microscope. For root cross‐sections, GUS‐stained seedlings were fixed and embedded in Historesin^TM^ (Leica Instruments GmbH; Wetzlar, Germany) and sectioned on a Leica microtome (Rodriguez‐Villalon *et al*., 2014). Sections were mounted in water and visualised with a 40× magnification objective of a compound microscope.

### Quantitative real‐time PCR gene expression analysis

For gene expression analysis, WT seedlings were grown on a nylon mesh on top of normal agar medium plates. After 5 days, seedlings were transferred via nylon mesh to Petri dishes containing incubation buffer and left for 2 h to recover. Seedlings were then treated for indicated times; samples were collected of ~50 seedlings in 2 ml safe‐lock Eppendorf tubes and immediately frozen in liquid nitrogen. Total RNA extraction and cDNA synthesis were performed, as indicated previously. qRT‐PCR was performed using HOT FIREPol EvaGreen qPCR mix Plus (ROX) (Solis Biodyne, Tartu, Estonia) and the ABI 7500 Real‐Time PCR system (Applied Biosystems, Waltham, MA, USA). The following PCR conditions were used: 50°C 2 min, 95°C 15 min, followed by 45 cycles of 95°C 15 sec and 60°C 1 min. Primers used for gene expression analyses are listed in Table S1. qRT‐PCR analyses were performed on three biological replicates with two technical replicates, using two reference genes, namely *SAND* (*At2g28390*) and *EXPRS* (*At2g32170*), which were shown to be very consistently expressed in different conditions by Hong *et al.* (2010).

### Confocal laser scanning microscopy

Arabidopsis transgenic lines containing the *Pro_Ubi10_:YFP‐PH_PLCδ1_* construct were grown for 5 days and then transferred for 30 min to small Petri dishes containing different treatments. Seedlings were then rinsed briefly with buffer, stained for 5 min with 2 µm FM4‐64 (Invitrogen), rinsed again twice with buffer and then mounted on a microscopy slide for analysis with a Zeiss LSM510 confocal microscope. eYFP and FM4‐64 were synchronously excited at 488 nm and 561 nm, respectively, and imaged using an HFT 405/488/561 nm major beam splitter and a 505 to 550 nm band‐pass filter and a 650 nm long‐pass filter, respectively. Images were converted to 8‐bit in ImageJ (www.imagej.net) for better visualisation of the eYFP and FM4‐64 signals. Plot profile analysis was performed and the region of interest based on single cells was selected for colocalisation measurements in ImageJ.

### Ion flux measurement

Net K^+^ flux was measured using non‐invasive MIFE (Shabala *et al*., 2006; Zarza *et al*., 2019). Five‐day‐old seedlings were immobilised in a 30 ml measuring chamber containing basic salt medium (BSM; 0.5 mm KCl, 0.2 mm CaCl_2_, 5 mm MES, 2 mm Tris base, pH 6.0). Roots were immobilised in a horizontal position (Bose *et al*., 2014) and pre‐incubated in BSM for at least 30 min. Electrodes were positioned 40 µm from the root surface in the elongation zone (less than 2 mm from the root cap junction). First, steady‐state ion fluxes were recorded over a period of 5 min; thereafter Spm was added and the net ion flux was measured for another 30 min.

### Root phenotyping assay on plates

Arabidopsis seedlings were grown on vertical plates containing standard sterile growth medium for 5 days and then transferred to medium supplemented with KCl or 0.22 µm filter‐sterilised Spm. Plates were scanned 4 days after transfer (4 DAT) using an Epson Perfection V700 Scanner at 300 dpi resolution. Root measurements were performed using EZ‐Rhizo software (Armengaud *et al*., 2009). Main root growth was expressed as growth ratio (MR_length_/MR_length_ at 0 DAT).

### Statistical analysis

SPSS was used for statistical analysis. For paired comparisons, the Student–Newman–Keuls test at *P* < 0.05 was used, were different letters indicate significantly different values. Student’s *t*‐test was used in comparisons with control treatments, where asterisks indicate significant differences: *P < 0.05, **P < 0.01, ***P < 0.005. Data shown represent the mean ± SD. The results obtained were confirmed by at least three independent experiments unless otherwise indicated.

## ACCESSION NUMBERS

*PIP5K7* (At1g10900); *PIP5K9* (At3g09920); *SAND* (At3g28390); *EXPRS* (At2g32170).

## AUTHOR CONTRIBUTIONS

XZ, SS, ARV and TM designed the experiments. LS performed MIFE analyses, AH GUS cross‐sectioning, ML phenotyping and XZ the rest, with assistance of RvW. ARV, AT, SS and IH added materials, ideas and discussions. XZ, IH and TM wrote the manuscript.

## CONFLICTS OF INTEREST

The authors declare that they have no conflict of interests.

## Supporting information


**Table S1**. Oligonucleotides used in this work.
**Figure S1**. Spm‐induced PIP_2_ response is not caused by breakdown products of Spm.
**Figure S2**. PA and PIP_2_ responses occur simultaneously.
**Figure S3**. Spm‐induced PIP_2_ responses in Arabidopsis *PIP5K* T‐DNA insertion mutants.
**Figure S4**. PIP_2_ levels in the *pldδ* mutant.
**Figure S5**. PIP_2_ levels in response to Spm in seedlings and mature leaves.
**Figure S6**. No obvious phenotypes are apparent in *pip5k7 pip5k9* mutants.
**Figure S7**. PIP_2_ levels are higher in SPMS overexpression lines.
**Data S1**. Supplementary methods.

## Data Availability

All data generated or analysed during this study are included in the article and its supplementary information files. Plasmids and mutants are available upon request.
