# Supplementary material for: Lipid kinases PIP5K7 and PIP5K9 are required for polyamine‐triggered K+ efflux in Arabidopsis roots
Source: Plant J. 2020 Aug 19;104(2):416–32. doi: 10.1111/tpj.14932 (PMC7693229; doi:10.1111/tpj.14932)
Supplement: Supplementary file 1 — Table S1. Oligonucleotides used in this work. Figure S1. Spm‐induced PIP2 response is not caused by breakdown products of Spm. Figure S2. PA and PIP2 responses occur simultaneously. Figure S3. Spm‐induced PIP2 responses in Arabidopsis PIP5K T‐DNA insertion mutants. Figure S4. PIP2 levels in the pldδ mutant. Figure S5. PIP2 levels in response to Spm in seedlings and mature leaves. Figure S6. No obvious phenotypes are apparent in pip5k7 pip5k9 mutants. Figure S7. PIP2 levels are higher in SPMS overexpression lines. Data S1. Supplementary methods. [file TPJ-104-416-s001.pdf]

SUPPORTING INFORMATION - Zarza *et al.* 2020

SUPPLEMENTAL METHODS

Treatment with ROS- and NO scavengers

$^{32}P_i$-prelabelled 6-days old seedlings were pre-treated with 5 mM N,N'-Dimethylthiourea (DMTU or 0.1 mM cPTIO for 60 min, and subsequently treated with buffer ± 120 µM Spm for 30 min (Zarza et al., 2019), and the PIP_2 response determined accordingly, as described above.

Microscopy and histological analysis

For the analysis of the vasculature differentiation in roots, 5-day-old seedlings were stained with propidium iodide (10 µg/ml) and imaged using a Zeiss LSM780 confocal microscope. For the analysis in cotyledons, 7-day-old seedlings were fixed and clarified as described by Carland and Nelson (2004), and imaged using a Zeiss AxioZoomV16 microscope.

SUPPLEMENTAL TABLE

Table S1. List of oligonucleotides used in this work.

Identification of homozygous *pip5k7-1*, *pip5k7-3*, *pip5k9a* and *pip5k9c* mutants

pip5k7-1 primer Fwd:	5'-TGCTATGTCGCAGAAACAATG-3'
pip5k7-1 primer Rev:	5'-TAAGTCAAACATGCCCCATTC-3'
pip5k7-3 primer Fwd:	5'-TGCAGTATGATCCGATGACAA-3'
pip5k7-3 primer Rev:	5'-CCATGATAGGAGGCAAATTGA-3'
pip5k9a primer Fwd:	5'-AATCAGTTCCATGTCGTCTGG-3'
pip5k9a primer Rev:	5'-GGTTCTTCTGAGGATGCTTCC-3'
pip5k9c primer Fwd:	5'-AGCAAGTTCTTGGAAGCACAG-3'
pip5k9c primer Rev:	5'-GGATCGAAAGCTCTTGGAAAC-3'
T-DNA (Left Border):	5'-ATTTTGCCGATTTCGGAAC-3'

Semi-quantitative RT-PCR analyses

AtPiP5K7_sqRT-PCR_Fwd:	5'-GGAGTTCACAGAATAACCCTC-3'
AtPIP5K7_sqRT-PCR_Rev:	5'-CCATGATAGGAGGCAAATTGA-3'
AtPiP5K9_sqRT-PCR_Fwd:	5'-GGTTCTTCTGAGGATGCTTCC-3'
AtPIP5K9_sqRT-PCR_Rev:	5'-TTTATTGAAGCTCTCAAGATCTGTAT-3'
SAND_sqRT-PCR_Fwd:	5'-CAGACAAGGCGATGGCGATA-3'
SAND_sqRT-PCR_Rev:	5'-GCTTTCTCTCAAGGGTTTCTGGGT-3'

Quantitative RT-PCR expression analyses

AtPiP5K7_qqRT-PCR_Fwd:	5'-CACAAGGTGTTCCCAGAAGAA-3'
AtPIP5K7_qRT-PCR_Rev:	5'-CCATGATAGGAGGCAAATTGA-3'
AtPiP5K9_qRT-PCR_Fwd:	5'-AGCCGTTGATCCAACATTCT-3'
AtPIP5K9_qRT-PCR_Rev:	5'-TTTATTGAAGCTCTCAAGATCTGTAT-3'
SAND_qRT-PCR_Fwd:	5'-CAGACAAGGCGATGGCGATA-3'
SAND_qRT-PCR_Rev:	5'-GCTTTCTCTCAAGGGTTTCTGGGT-3'

Amplification of GUSPlus gene from the pCAMBIA 1305.1 vector

5'- GATCGCGGCCGCCATGGTAGATCTGAGGGTAAATTTCTAGTTTTTCTC-3'
5'-GATCGAGCTCCTGTCAAACACTGATAGTTTAATTCCCGATC-3'

Amplification of *PIP5K7* and *PIP5K9* promotors

PromPIP5K7:	5'-GATCGTCGACGGTCGGGCTTTTTTATTTATTCAGCGATATCTG-3'
BAC clone T19D16:	5'-GATCGCGGCCGCCTGGGATTAGCTTTTGGTGATCCTAGAACATTG-3'
PromPIP5K9:	5'-GATCGTCGACGGTGAGCAGAAGTTGACTTAAAAATATTGG-3
BAC clone F8A24:	5'-GATCGCGGCCGCGTAAATCTCCAAAGAAGCTAAAGCAGAAAC-3'

SUPPLEMENTAL FIGURES

Supplemental Figure S1 – Zarza *et al*.

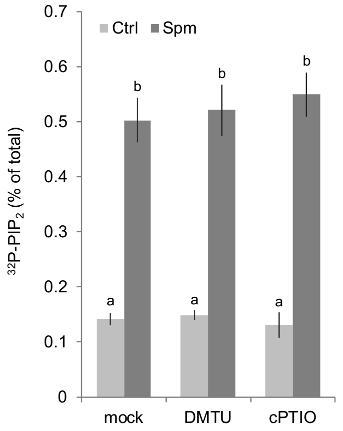

Figure S1. Spm induced-PIP$_2$ response is not caused by breakdown products of Spm. Quantification of PIP$_2$ levels in ^{32}P-labelled seedlings incubated in the presence of buffer (control; ctrl) or scavengers for 60 min, and then treated for 30 min with or without 120 µM Spm. Mean ± SD (n = 4).

Supplemental Figure S2 – Zarza *et al*.

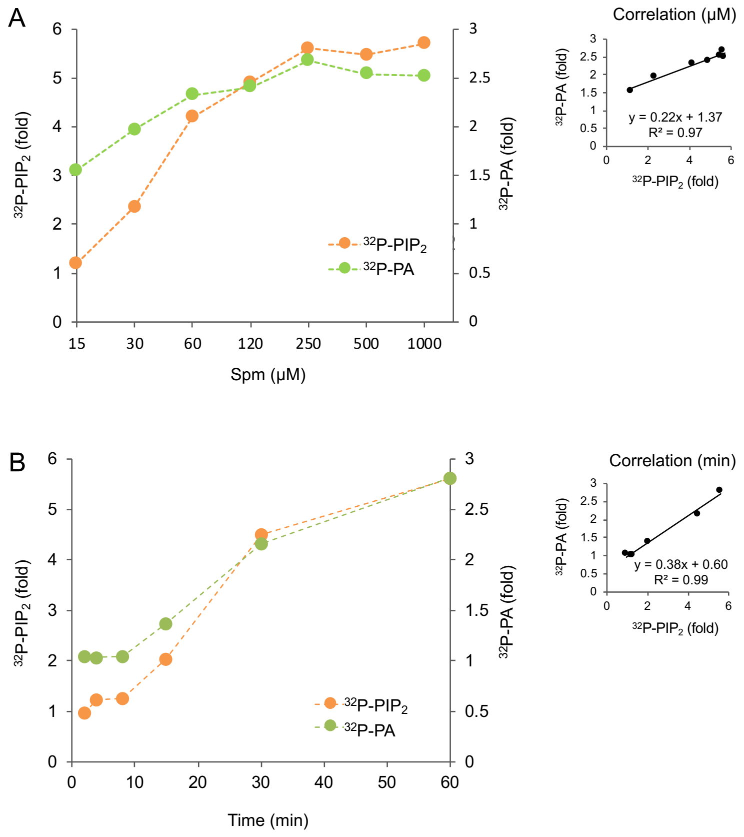

Figure S2. PA and PIP$_2$ responses occur simultaneously.

^{32}P$_i$-labelled seedlings were treated with Spm and the PA- and PIP$_2$ responses determined simultaneously. Fold-responses were calculated from control levels, i.e. buffer without Spm, taken as 1. (**A**) Dose-response of indicated Spm concentrations after 30 min. (**B**) Time course of responses using 60 µM Spm. For all data results are mean ± SD (n = 6).

Supplemental Figure S3 – Zarza *et al*.

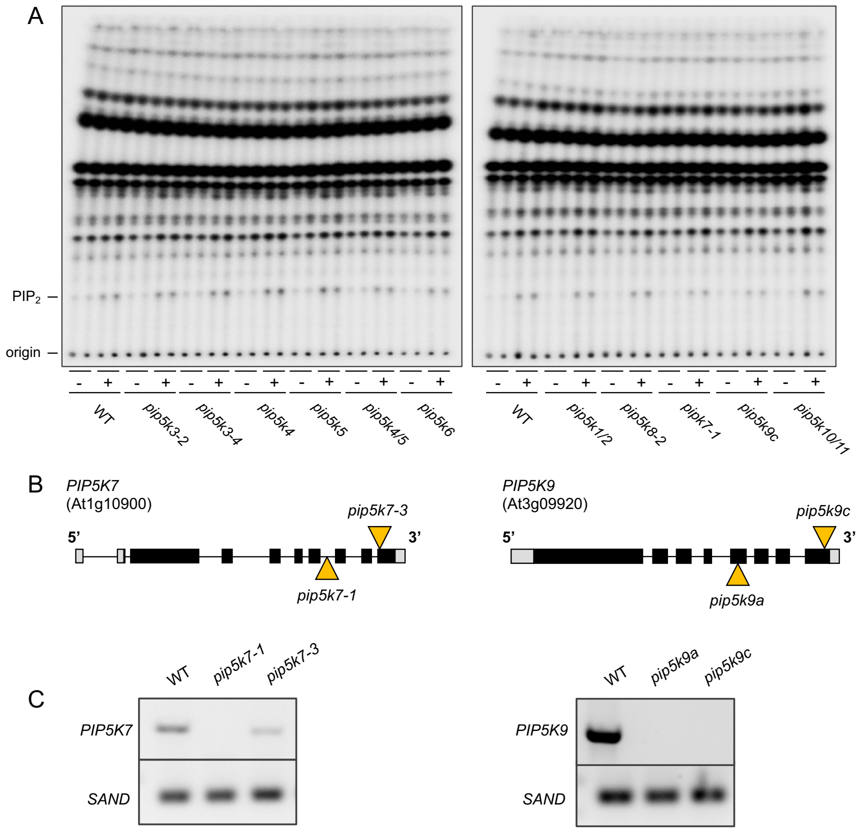

Figure S3. Spm induced-PIP₂ responses in Arabidopsis *PIP5K* T-DNA-insertion mutants. (**A**) Autoradiograph of TLCs showing ^{32}P-PIP$_2$ responses in PIP5K T-DNA insertion mutants that had been ^{32}P$_i$-labelled O/N and treated with (+) or without (-) 60 μM Spm for 30 min. Mutants analysed: *pip5k1* (SALK_146728), *pip5k2* (SALK_012487), *pip5k3-2* (SALK_001546), *pip5k3-4* (SALK_126683), *pip5k4* (SALK_001138), *pip5k5* (SALK_147475), *pip5k6* (SALK_096280), *pip5k7-1* (SALK_151429), *pip5k8-2* (SALK_040022), *pip5k9c* (SALK_013602), *pip5k10* (SALK_119243), *pip5k11* (GABI_284F05). (**B**) Schematic representation of the structure of the *PIP5K7*- and *PIP5K9* genes in *Arabidopsis*. Exons (black boxes), UTR exon (white boxes), introns (lines) and positions for the insertions in T-DNA alleles *pip5k7-1*, *pip5k7-3* (SALK_107796), *pip5k9c* and transposon allele *pip5k9a* (SM_3_39157) are shown. (**C**) semi-quantitative RT-PCR analysis of the insertion mutants, showing null transcript levels of *PIP5K7* for *pip5k7-1* and lower transcript levels for *pip5k7-3*, and null transcript levels of *PIP5K9* for both *pip5k9a* and *pip5k9c* lines. The Arabidopsis *SAND* gene was used as a positive internal control.

Supplemental Figure S4 – Zarza *et al.*

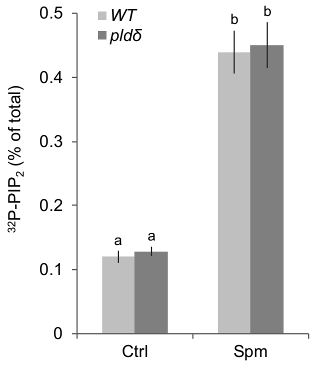

Figure S4. PIP_2 levels in *pldδ* mutant.
^{32}P-PIP_2 response in 5 days-old wt- and *pldδ* seedlings that were labelled O/N and treated with or without 60 µM Spm for 30 min. Mean ± SD (n = 4).

Supplemental Figure S5 – Zarza *et al*.

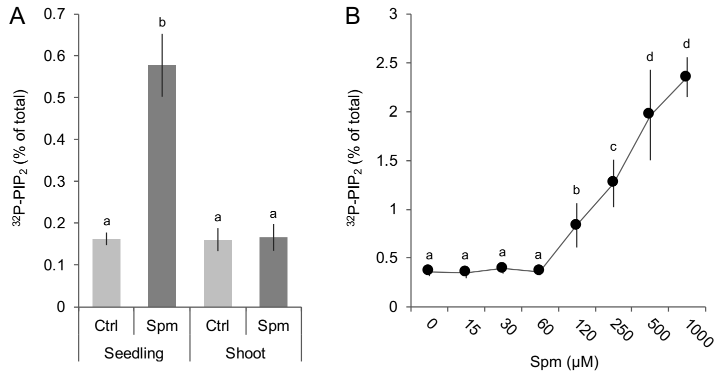

Figure S5. PIP$_2$ levels in response to Spm in seedlings and mature leaves.
In young seedlings, only a PIP$_2$ response in the root is triggered, not in shoots. In mature leaves, a PIP$_2$ response can be observed in $^{32}P_i$-prelabelled leaf discs, though higher concentrations were required than for seedlings. (**A**) ^{32}P-PIP$_2$ levels in roots and shoots of WT seedlings treated with or without 60 µM Spm for 30 min. (**B**) Leaf disks, punched from 3-weeks-old *Arabidopsis* plants, were labelled O/N and the next day treated for 30 min at indicated Spm concentrations, after which lipids were extracted, separated by TLC and quantified by phosphoimaging. Data are the mean ± SD (n = 9).

Supplemental Figure S6 – Zarza *et al*.

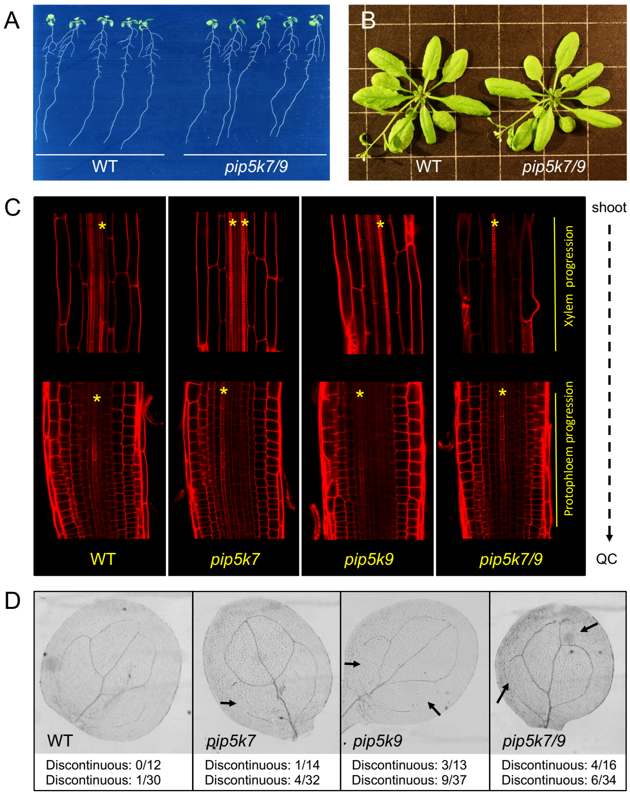

Figure S6. No obvious phenotypes are apparent in *pip5k7 pip5k9* mutants.
(**A**) Picture of 9-day-old seedlings grown on plates containing standard medium. (**B**) Representative shoot of 4 week-old plants grown on soil. (**C**) Confocal images of 5-day-old propidium-iodide stained (red) roots, displaying the xylem-differentiation region (upper panel) and protophloem-differentiation zone (lower panel). Yellow asterisks mark the corresponding vascular strand. (**D**) Cotyledon vascular patterning of 7-day-old seedlings of the indicated genotypes. Black arrowheads mark discontinuities in the vascular network. The ratio of seedlings from two independent experiments showing vascular network discontinuity in the cotyledons is indicated (n = 12-37).

Supplemental Figure S7 – Zarza *et al*.

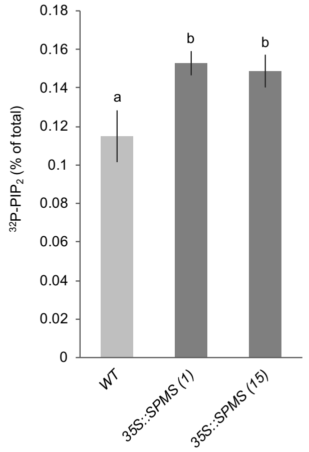

Figure S7. PIP_2 levels are higher in SPMS-overexpressor lines.
WT and two independent *Pro35S::SPMS* lines (#1, #15) were ^{32}P-labelled O/N. Next day, lipids were extracted and PIP_2 levels quantified. Mean ± SD (n = 3).
